# Vaginitis in the Context of an Uncommon Parasomnia: A Case Report

**DOI:** 10.7759/cureus.62696

**Published:** 2024-06-19

**Authors:** Mel Ebeling, Erin F Shufflebarger, Zachary Pacheco

**Affiliations:** 1 Emergency Medicine, University of Alabama, Birmingham Heersink School of Medicine, Birmingham, USA

**Keywords:** sexsomnia, case report, parasomnia, gynecological pathology, vaginitis

## Abstract

Sexsomnia is a rare, male-predominant, non-rapid eye movement parasomnia characterized by complex sexual behaviors occurring without conscious awareness during sleep. The biopsychosocial consequences of sexsomnia on both those diagnosed and their bed partners have not yet been fully elucidated. We present the case of an adult, a heterosexual female who developed vaginitis following sexual intercourse that occurred secondary to her partner’s diagnosed sexsomnia. To our knowledge, this is the first reported case of gynecological pathology occurring because of sexsomnia, and it serves to highlight the importance of thorough history-taking and the need for further research on the effects of sexsomnia on both parties involved.

## Introduction

Sexsomnia, colloquially termed “sleep sex,” is a non-rapid eye movement (NREM) parasomnia characterized by complex sexual behaviors such as masturbation, groping, fondling, and sexual intercourse that occurs without conscious awareness during sleep [1,2]. While the epidemiology of this disorder is not entirely known, affected individuals are predominantly male [3]. Unlike other NREM parasomnias like sleepwalking and sleep terrors, the existing literature on sexsomnia is largely limited to case series, and it was not until 2013 that sexsomnia was first legitimized by the American Psychological Association through its inclusion in the fifth edition of the Diagnostic and Statistical Manual of Mental Disorders (DSM-5) [2]. Given the nature of the disorder, sexsomnia inherently affects the person diagnosed in addition to their bed partners. Yet the ramifications of sexsomnia acknowledged in the literature seems to be confined to the psychosocial consequences of rape and sexual assault that have occurred because of the nonconsensual sexual behaviors imposed on the bed partner [1,3,4]. To our knowledge, bodily consequences of sexsomnia on the bed partner have not been previously described. Here, we present the novel case of a woman who developed vaginitis secondary to her partner’s diagnosed sexsomnia.

## Case presentation

A heterosexual, premenopausal adult female with no pertinent past medical history presented to the emergency department (ED) with a three-day course of dysuria, urinary frequency, pulling sensation in the suprapubic area, and rectal pain exacerbated by bowel movements. Upon further questioning, the patient disclosed that her sexual partner, a cisgender male with diagnosed sexsomnia, frequently engages in vaginal intercourse with her while they are both asleep, and she does not always become fully awake and alert during these occurrences. A few episodes of this sexual activity occurred within days of her presentation in close temporal proximity to the development of her symptoms. In addition to her symptoms, the patient also had concerns about a possible retained tampon as a result of the sexual intercourse that occurred while she was asleep and on her menstrual cycle.

A speculum examination was conducted and was without evidence of any retained foreign bodies, bleeding, cervical masses, or ulcerations, but was significant for malodorous discharge. Urinalysis was significant for positive leukocyte esterase and elevated red and white blood cell counts (Table 1). A urine pregnancy test was negative, and a complete blood count and basic metabolic panel were within normal limits. A diagnosis of vaginitis was made, given the foul-smelling odor appreciated while performing the pelvic exam in addition to her symptoms, and she was empirically treated for sexually transmitted infections (i.e., chlamydia, gonorrhea) and bacterial vaginosis with one dose of intramuscular ceftriaxone in the department alongside prescribed oral doxycycline and metronidazole. Following discharge from the ED, her urine culture was negative for bacterial growth but had a comment of probable contamination, and her gonorrhea and chlamydia cervical swab testing resulted as negative.

**Table 1:** Urinalysis findings

Parameter	Measured result	Unit	Reference range
Color	Yellow	-	-
Clarity	Clear	-	-
Specific gravity	1.029	-	1.003-1.035
pH	6.5	-	4.5-8
Protein	Negative	-	Negative
Ketones	Negative	-	Negative
Blood	Negative	-	Negative
Nitrite	Negative	-	Negative
Leukocyte esterase	2+	-	Negative
Red blood cells	11-20	/High-power field	0-2
White blood cells	26-50	/High-power field	0-5
Bacteria	Rare	-	Negative

## Discussion

We presented the case of a heterosexual adult cisgender female who developed vaginitis in temporal proximity to sexual intercourse that occurred as part of her male partner’s sexsomnia. To our knowledge, this is the first reported case of gynecological pathology occurring because of sexsomnia.

In the United States, vaginitis, or inflammation of the vagina, is a common gynecological diagnosis resulting in an estimated 10 million office visits each year [5]. Common causes of vaginitis include bacteria, perfumed soaps, protozoa (e.g., *Trichomonas vaginalis*), and yeast [6]. Sexual activity is a strong risk factor for the development of vaginitis [7,8].

While the gynecological pathology described in this case is not unique, the discussion of its development secondary to parasomnia is crucial. Because sexual intercourse increases the risk for vaginitis in cisgender females, sexsomnia may serve as a risk factor for the development of this condition. When recurrent, vaginitis is associated with an increased risk of tubal factor infertility [9]. Thus, while this condition is typically easy to diagnose and treat, thorough history-taking to assess risk factors is an important aspect of the prevention of recurrence. Bed partners may be fearful or embarrassed to report their symptoms in relation to their partner’s sexsomnia, as in this case. Additionally, it is within the realm of possibility for an individual with undiagnosed sexsomnia to have symptoms of vaginitis and not be aware of their behaviors, placing them at risk. In cases of recurrent vaginitis or unexplained genital trauma in sexually active women or recurrent urethritis or prostatitis in sexually active men without underlying structural abnormalities and in which other risk factors have been modified or ruled out, asking the patient and ideally their partners, if possible, about sexsomnia behaviors should be considered. In patients, such as ours, who are aware of their partner’s diagnosis, environment modification (e.g., sleeping in separate beds or rooms) and treatment of the parasomnia, which has previously been attempted with clonazepam and good sleep hygiene, may be beneficial in preventing future recurrences of vaginitis.

## Conclusions

This case report highlights sexsomnia as a rare but possible risk factor for the development of gynecological pathology in bed partners of those with the disorder. More research is ultimately necessary to fully characterize the biopsychosocial consequences of sexsomnia on both bed partners and those diagnosed. In the meantime, clinicians should ensure a thorough history is obtained from all patients presenting with symptoms of typically uncomplicated and straightforward gynecological pathology to prevent unnecessary recurrences. A high degree of suspicion for uncommon etiologies of gynecological pathology like sexsomnia should be maintained when individuals present with vaginitis without any other identifiable behavioral, functional, or anatomical cause. When sexsomnia is suspected in a patient or bed partner, education and referral to a sleep medicine physician for further evaluation should be provided.
